# Paracentral Acute Middle Maculopathy As the Presenting Sign of Ischemic Cardiomyopathy

**DOI:** 10.7759/cureus.35418

**Published:** 2023-02-24

**Authors:** Priyadarshini Mishra, Satyapriya Mohanty, Shanmugasundaram P, Bruttendu Moharana, Debasish Das

**Affiliations:** 1 Ophthalmology, All India Institute of Medical Sciences, Bhubaneswar, IND; 2 Cardiothoracic Surgery, All India Institute of Medical Sciences, Bhubaneswar, IND; 3 Cardiology, All India Institute of Medical Sciences, Bhubaneswar, IND

**Keywords:** ischemic cardiomyopathy, central retinal vein occlusion, amn, pamm, acute macular neuroretinopathy, macular oct, parafoveal lesion, macular ischemia, paracentral acute middle maculopathy

## Abstract

Paracentral acute middle maculopathy (PAMM) is a type of ischemic maculopathy affecting intermediate and deep retinal capillary plexuses. A typical presentation is acute onset scotoma with or without vision loss. It is characterized by greyish-white parafoveal lesions. Sometimes very subtle lesions can be missed on clinical examination. The main diagnostic modality is spectral domain optical coherence tomography (SD-OCT) wherein focal or multifocal lesions are seen as bands of hyperreflectivity in the inner nuclear and outer plexiform layers. This entity can be associated with systemic microvascular diseases. Here, we report an interesting case of PAMM as the only presenting sign in a patient with ischemic cardiomyopathy, highlighting the necessity for a thorough systemic examination in such patients.

## Introduction

Paracentral acute middle maculopathy (PAMM) represents a type of ischemic maculopathy. This entity was first described by Sarraf et al. in 2013 as a variant of acute macular neuroretinopathy (AMN) [1]. Subsequently, these lesions have been associated with a multitude of retinal vascular diseases including branch and central retinal arterial occlusion, central retinal vein occlusion (CRVO), diabetic retinopathy, sickle cell retinopathy, and Purtscher retinopathy. It can also be seen after a flu-like illness, and transient orbital compression [2]. This wide spectrum of associated conditions has prompted us to consider PAMM as a clinical sign and not a separate disease entity. Extensive investigations are required to rule out all the systemic risk factors.

A typical presentation is a sudden onset paracentral scotomas with or without decreased vision, parafoveal greyish-white lesions, and hyperreflective bands localized to the middle layers of the retina, mainly the outer plexiform layer (OPL) and inner nuclear layer (INL) on spectral domain optical coherence tomography (SD-OCT). The characteristic lesions are due to ischemia of the intermediate and deep capillary plexuses [3].

This condition can be associated with systemic microvascular diseases. We report a case of PAMM as the only presenting sign of underlying ischemic cardiomyopathy in an otherwise healthy male.

## Case presentation

A 50-year-old male presented with acute onset scotoma in the right eye. He had no other ocular or systemic symptoms. No history of any systemic disease, prolonged medication, headache, or trauma was elicited.

**Figure 1:**
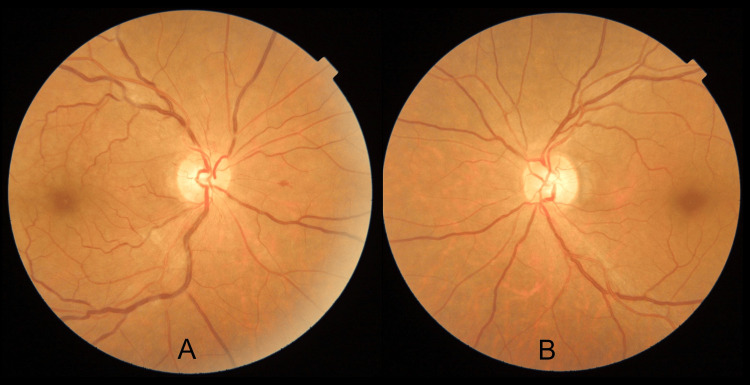
Fundus photograph A: Fundus photograph of the right eye showing mild blurring of superior and inferior disc margin, dilated and tortuous vein, arterial attenuation, A-V crossing changes, and one superficial hemorrhage on the nasal side of the disc B: Fundus photograph of the left eye showing a normal disc, healthy macula, arterial attenuation, and A-V crossing changes

On examination, his best corrected visual acuity was 6/6 in both eyes. Anterior segment findings were unremarkable. On dilated fundus examination of the right eye, mild blurring of the superior and inferior disc margin, dilated and tortuous vein, arterial attenuation, A-V crossing changes, and one superficial hemorrhage were noticed. In the macula, subtle greyish-white parafoveal lesions were suspected but it was not very clear. A provisional diagnosis of grade 3 hypertensive changes with impending CRVO was made. In the left eye, a normal disc, healthy macula, arterial attenuation, and A-V crossing changes were suggestive of hypertensive retinopathy grade 2 (Figure 1 A, B)

**Figure 2:**
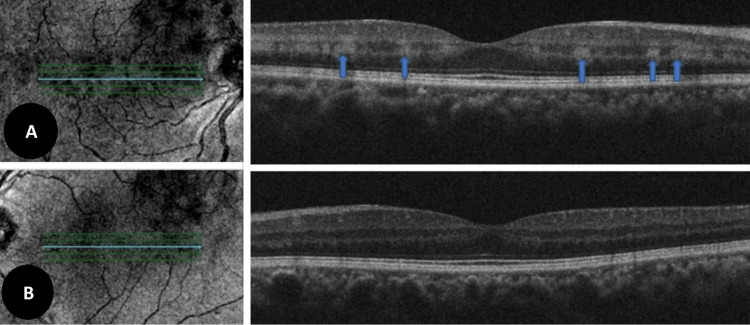
SD-OCT scan of macula A: Right eye OCT (HD raster macula) showing localized hyperreflective bands at the level of inner nuclear-outer plexiform layer (blue arrows) and corresponding near-infrared reflectance image (upper panel) B: OCT and near-infrared reflectance of the left eye showing healthy macula (lower panel) SD-OCT: Spectral domain optical coherence tomography

The patient was subjected to further ocular and systemic investigations. On SD-OCT macular scan, localized hyperreflective bands at the level of the inner nuclear-outer plexiform layer were noticed in the right eye whereas the left eye scan was normal (Figure 2 A, B).

**Figure 3:**
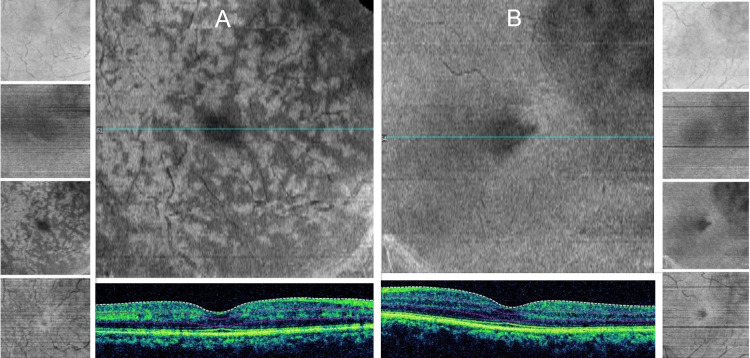
En-face OCT A: En-face OCT analysis of macular cube 512×128 of the right eye; parafoveal lesions are clearly visible as hyporeflective patches in mid-retina B: En-face analysis of the left eye shows a normal macula

Parafoveal lesions which were very subtle clinically became visible in the en-face OCT of the right eye as multiple perivenular fern-like hyporeflective patches were visible at the level of the mid-retina compared to normal left eye scan (Figure 3 A, B).

**Figure 4:**
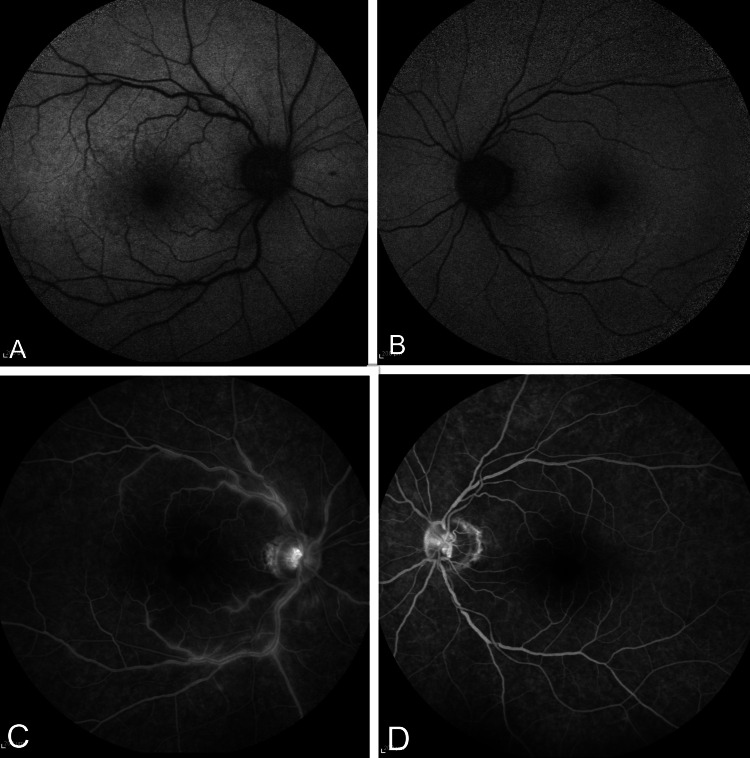
Fundus autofluorescence and fundus fluorescein angiography (FFA) In fundus autofluorescence of the right eye (A), multiple hypoautofluorescent parafoveal lesions are evident compared to the healthy macula of the left eye (B). The late phase of FFA of the right eye (C) shows staining of retinal veins. No leakage from the disc or macula is seen. The FFA of the left eye (D) shows normal staining of the optic disc.

**Figure 5:**
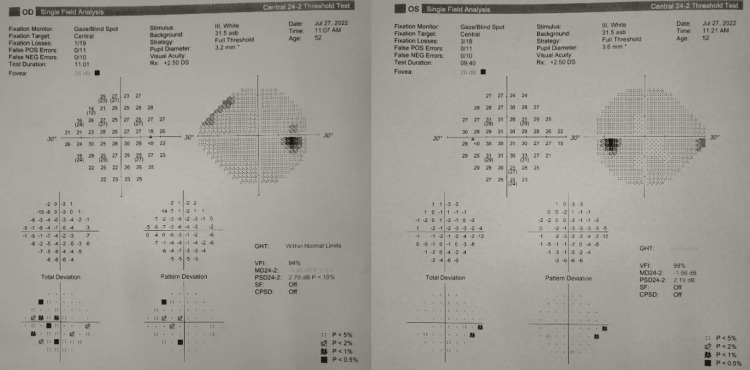
Visual field test Visual field testing demonstrated a focal loss in the parafoveal area of the right eye

In the fundus autofluorescence image, hypoautofluorescent spots were noted in the parafoveal area of the right eye whereas the left eye showed a healthy macula. Fundus fluorescein angiography (FFA) in the right eye showed delayed arm-to-retina circulation time, prolonged arteriovenous transit time, and late staining of retinal veins. No leakage from the disc or macula was seen. The FFA was normal in the left eye (Figure 4 A, B, C, D). Visual field testing demonstrated a focal loss in the parafoveal area of the right eye (Figure 5).

**Figure 6:**
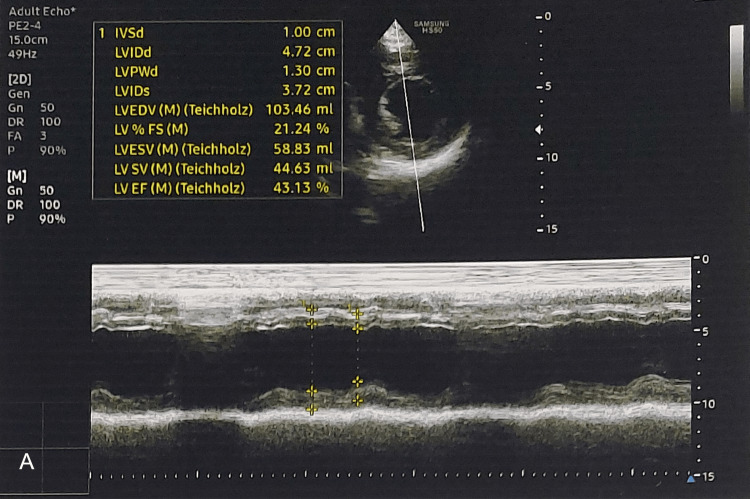
Echocardiography The echocardiography shows global hypokinesia of the left ventricle with moderate systolic dysfunction, mild mitral regurgitation, and an ejection fraction of 43%.

**Figure 7:**
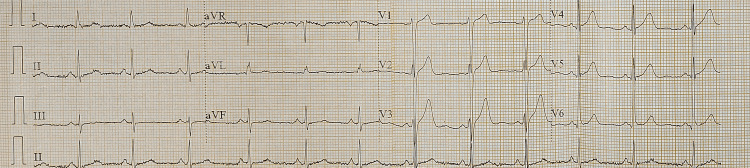
Electrocardiogram The ECG shows poor R-wave progression (V3)

From all these ancillary tests, a diagnosis of PAMM was confirmed along with impending CRVO in the right eye. On routine investigations, hypertension and hyperlipidemia were detected. A COVID-19 reverse transcription-polymerase chain reaction (RT-PCR) and systemic inflammatory markers were negative. The patient was referred for a detailed cardiological evaluation. Echocardiography showed global hypokinesia of the left ventricle with moderate systolic dysfunction, mild mitral regurgitation, ejection fraction of 43% (Figure 6). The ECG demonstrated poor R-wave progression (V3), which was thought to be suggestive of ischemic cardiomyopathy (Figure 7). Coronary angiography further confirmed the diagnosis.

## Discussion

Sarraf et al. first interpreted PAMM as a possibly more superficial variant of AMN [1]. But subsequently, a multitude of systemic and retinal microvascular diseases demonstrated this clinical sign. Vasculopathic risk factors, such as arterial hypertension, dyslipidemia, carotid disease, or diabetes should be screened whenever PAMM is suspected. Paracentral acute middle maculopathy can be an isolated finding or can be seen with other signs of branch and central retinal arterial occlusion, CRVO, diabetic retinopathy, sickle cell retinopathy, and Purtscher retinopathy [2,4]. Paracentral acute middle maculopathy is thought to be a cause of decreased vision in non-ischemic CRVO [5,6].

Situated deeper within the retina in the parafoveal area, PAMM lesions are greyish-white, unlike cotton wool spots. Sometimes clinical appearance can be quite subtle, which can be detected by funduscopic examination. Its diagnosis relies on multimodal imaging such as SD-OCT, en-face OCT, fundus autofluorescence, and OCT-angiography.

The main differential diagnosis is AMN. The OCT is invaluable in confirming the diagnosis of PAMM as band-like multiple or isolated hyperreflectivity in the OPL-INL whereas AMN displays hyperreflectivity of the OPL and outer nuclear layer (ONL) and may be associated with disruption of the ellipsoid zone (EZ) [1]. The OCT angiography (OCTA) shows reduced flow in the intermediate retinal capillary plexus (ICP) and deep capillary plexus (DCP) in PAMM, whereas AMN is associated with reduced flow in the DCP only [7,8]. Three distinct patterns of these lesions were observed in en-face OCT: arteriolar, globular, and perivenular fern-like [1,9,10].

Paracentral acute middle maculopathy lesions regress over time. In most patients scotoma usually persists. On OCT, the hyperreflective band disappears within months and thinning and irregularity of the middle retinal layers specially INL and OPL are noted [1,10]. Excavated change of the inner retinal surface, along with outer nuclear layer (ONL) thickening can also be seen [11]. On OCTA resolution, capillary dropout is noted leaving disorganization of the deep capillary plexus [11]. Fundus fluorescein angiography does not add much information to PAMM lesions but is helpful to rule out ischemic damage due to other associated conditions.

Paracentral acute middle maculopathy can be the sole manifestation of an underlying systemic microvascular disease in an apparently healthy person as in our case. So, an appropriate systemic work-up should be done to exclude all cardiovascular risk factors. Timely intervention can decrease mortality.

## Conclusions

Diagnosis of PAMM should increase the index of suspicion for ischemic cardiovascular disease, the timely management of which can improve long-term outcomes. Clinical signs can be very subtle sometimes and can be detected via fundoscopy examination. The typical history of acute onset paracentral scotoma and multimodal imaging findings will aid this diagnosis. 
